# Quantifying changes in respiratory syncytial virus—associated hospitalizations among children in Texas during COVID-19 pandemic using records from 2006 to 2021

**DOI:** 10.3389/fped.2023.1124316

**Published:** 2023-03-13

**Authors:** Inyang Uwak, Natalie Johnson, Toriq Mustapha, Mariya Rahman, Tanaya Tonpay, Annette K. Regan, Itza Mendoza-Sanchez

**Affiliations:** ^1^ Department of Environmental & Occupational Health, Texas A&M University School of Public Health, College Station, TX, United States; ^2^ School of Nursing and Health Professions, University of San Francisco, San Francisco, CA, United States

**Keywords:** RSV, Texas, hospital admissions, length of hospital stay, COVID-19

## Abstract

**Aim:**

To quantify changes on RSV- associated hospitalizations during COVID-19 pandemic, among children four years of age or younger at the state and county levels of Texas using routinely acquired hospital admission records.

**Methods:**

We used the Texas Public Use Data Files (PUDF) of the Department of State Human Services (DSHS) to obtain hospital admissions and healthcare outcomes from 2006 to 2021. We used the 2006–2019 period to estimate a long-term temporal trend and predict expected values for 2020–2021. Actual and predicted values were used to quantify changes in seasonal trends of the number of hospital admissions and mean length of hospital stay. Additionally, we calculated hospitalization rates and assessed their similarity to rates reported in the RSV Hospitalization Surveillance Network (RSV-NET).

**Results:**

An unusually low number of hospitalizations in 2020 was followed by an unusual peak in the third quarter of 2021. Hospital admissions in 2021 were approximately twice those in a typical year. The mean length of hospital stay typically followed a seasonal trend before COVID-19, but increased by a factor of ∼6.5 during the pandemic. Spatial distribution of hospitalization rates revealed localized healthcare infrastructure overburdens during COVID-19. RSV associated hospitalization rates were, on average, two times higher than those of RSV-NET.

**Conclusion:**

Hospital admission data can be used to estimate long-term temporal and spatial trends and quantify changes during events that exacerbate healthcare systems, such as pandemics. Using the mean difference between hospital rates calculated with hospital admissions and hospital rates obtained from RSV-NET, we speculate that state-level hospitalization rates for 2022 could be at least twice those observed in the two previous years, and the highest in the last 17 years.

## 1. Introduction

Respiratory syncytial virus (RSV) is a common airway pathogen that most frequently results in mild, cold-like respiratory tract infections. However, in children younger than two years of age RSV infection can result in severe lower respiratory illness, including acute bronchiolitis or pneumonia (1). In the U.S., an estimated 58,000 children younger than five years of age require hospitalization for RSV infection every year (2). Severe infection risk is highest in premature, low birth weight and young infants (<6 months old), and children with preexisting conditions, such as congenital heart disease (3, 4). Environmental factors, such as crowded conditions, daycare attendance, and exposure to tobacco smoke or air pollutants can also increase individual RSV infection risk (4).

The U.S. Centers for Disease Control and Prevention (CDC) evaluates seasonal trends using the National Respiratory and Enteric Virus Surveillance System (NREVSS) and the RSV hospitalization surveillance network (RSV-NET). The NREVSS employs a voluntary laboratory-based surveillance system, with data reported by sentinel laboratories on a weekly basis. The RSV-NET is a network that conducts active surveillance of children younger than 18 years of age and adults, and covers 8% of the U.S. population in 58 counties of the 12 states that participate in the Emerging Infections Program (5). While NREVSS and RSV-NET data are useful in characterizing RSV seasonality, especially at the national level, they may not always reflect RSV related hospital burden at the state or county level. Routinely collected data on healthcare service utilization, an alternative to surveillance systems, can be used to retrospectively investigate RSV epidemiology and healthcare outcomes at different temporal and spatial scales (6–10).

Season is a strong predictor of RSV infection, with activity typically occurring in the late fall, winter, and early spring, and peaking from late December to mid-February (11). The COVID-19 pandemic has, however, recently altered RSV seasonality. A reduction of cases was observed during 2020, followed by a delayed peak in the spring and summer of 2021 and, in some cases, higher-than-expected infection levels (10, 12–17). The main objective of this study was to quantify the impact of the COVID-19 pandemic on pediatric RSV infections at the state and county level using hospital admissions data from the state of Texas. For this, we used the number of hospitalizations and healthcare outcomes from 2006 to 2019 to predict expected values for 2020–2021, and quantify changes in seasonal trends, number of hospital admissions and mean length of hospital stay. A second objective was to calculate hospitalization rates with hospital admission data and assess if they were similar to rates reported in the RSV Hospitalization Surveillance Network (RSV-NET) from the Centers for Disease Control and Prevention (CDC).

## 2. Materials and methods

### 2.1. Data, RSV cases, and healthcare outcomes

**Figure 1:**
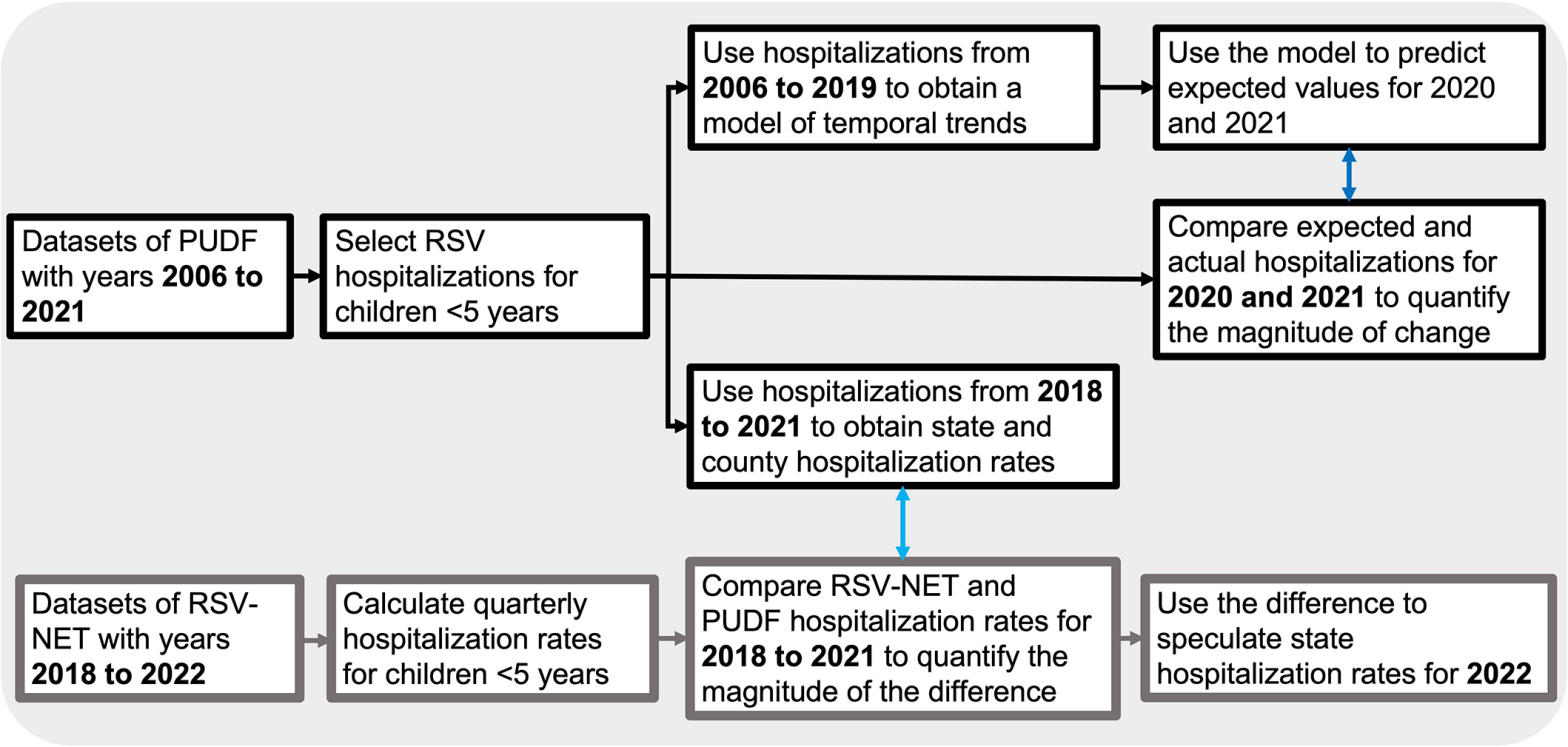
Graphical description of the methodology.

The datasets used in this study are Texas hospital discharge records from the Texas Public Use Data Files (PUDF) arranged each year by quarter (18). The Department of State Human Services (DSHS) Center for Health Statistics is responsible for collecting and releasing the data. The PUDF contains data on discharges (167 variables) from all state licensed hospitals except those that are statutorily exempt from the reporting requirement (18). The variables used for our analyses were: discharge diagnoses, length of stay, patient age, patient status at the end of service and patient county. The analysis included datasets from 2006 quarter 1 (2006 Q1) to 2021 quarter 4 (2021 Q4). RSV-associated hospitalization was defined as any admission with an ICD-9-CM or ICD-10-CM disease code consistent with RSV infection (Supplementary Table S1). We included all discharge diagnoses in the dataset, including admitting, principal, and 24 additional discharge diagnoses. From 2006 to 2015 Q3, when the ICD-9-CM coding system was used, RSV cases included RSV, acute bronchiolitis due to RSV, and pneumonia due to RSV. From 2015 Q4 to 2021Q4, when the ICD-10-CM coding system was used, RSV cases included RSV as the cause of diseases classified elsewhere, acute bronchitis due to RSV, acute bronchiolitis due to RSV, and RSV pneumonia. We restricted our analysis to pediatric RSV-associated hospitalizations of patients whose age at admission was <5 years old. We used variables describing patient status at discharge and the length of hospital stay to assess healthcare outcomes. Quarterly hospitalization rates were calculated using the population of patients aged birth through four years of age at the state and county levels as the denominator. Population projections for each year (2006 to 2021) based on the 2000 and 2010 censuses were obtained from the Texas Demographic Center. Spatial distribution maps of quarterly RSV infection rates at the county level were generated using ArcGIS 10.8.1 (ESRI, California, United States). To evaluate how the RSV associated hospitalization rates calculated in this study compare to RSV associated hospitalization rates reported in the RSV-NET from the CDC, we calculated cumulative quarterly hospitalization rates for children four years of age or younger from monthly RSV associated hospitalization rates reported in the RSV-NET from 2018 to 2022. A graphical description of the methodology is depicted in Figure 1.

### 2.2. Statistical analysis

For each quarter between 2006 and 2019, we generated a linear model of hospital admissions over time, and used the model to predict the number admissions for each quarter from 2020 to 2021 assuming no seasonal changes occurred. We compared observed and predicted hospital admissions for 2020 and 2021 and evaluated seasonal changes. A similar analysis was carried out for the length of hospital stay. We used the mean length of stay in each quarter to obtain a linear model for years 2006 to 2019, and evaluated changes in 2020 and 2021. We conducted the Wilcoxon Mann-Whitney test for non-normally distributed data to assess if the difference between the mean length of hospital stay for patients in 2019 and 2020 was statistically significant. In all analyses, we used R v.4.2.1 language functions, as described in detail in each section.

## 3. Results

A total of 158,920 RSV-associated hospitalizations were identified between 2006 and 2021. Most RSV associated hospitalizations from 2006 to 2019 were for children under five years of age (∼89%), though this proportion was lower in 2020 (∼66%) and 2021 (∼80%) (Supplementary Table S2). In the PUDF, records of children under 5 years were reported in three categories, 1–28 days old, 29–365 days old, and 1–4 years old. From 2006 to 2019, the highest percentage of hospital admissions was observed in the group of 29–365 days of age (65%), and the lowest in the group of 1–28 days of age (8%), which was consistent across quarters (Supplementary Table S3). The distribution of patient age at admission was similar in 2020, but in 2021 the highest percentage shifted to patients 1–28 days of age, followed by 1–4 years (on average 57% and 40% respectively) (Supplementary Table S3).

**Figure 2:**
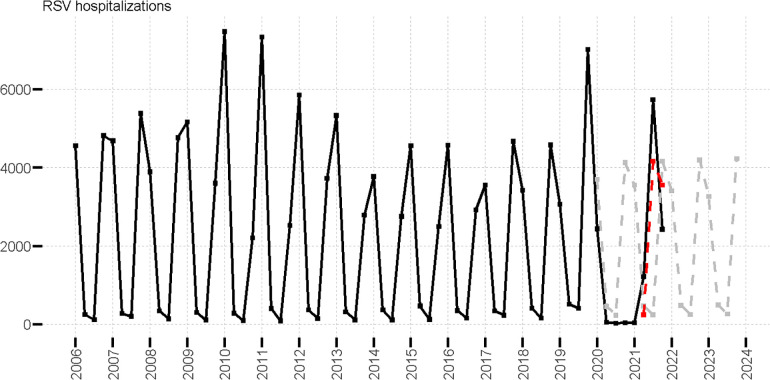
Observed RSV hospitalizations in patients aged four years and younger (black line), predicted RSV hospitalizations (gray dotted line), and predicted delayed RSV hospitalizations (red dotted line) values.

Seasonal patterns of hospital admissions in patients aged four years and younger show a consistent trend between 2006 and 2019. Observed values are shown in Figure 2, where seasonal peaks are observed in Q4 (Oct-Dec) and Q1 (Jan-Mar), and lower hospital admission levels in Q2 (Apr-Jun) and Q3 (July-Sept). The highest number of cases was observed in the 2010, 2011 and 2019 seasons (Figure 2). From 2006 to 2019 a general decreasing trend was observed for cases in Q1, with an increasing trend for all other quarters (Supplementary Table S4 and Figure S1). We generated linear regression models for each quarter using data from 2006 to 2019 (Supplementary Information) to predict hospital admissions for 2020 to 2021 assuming that COVID-19 had no impact on seasonality (observations and predictions are illustrated in Figure 2). An unusually low number of hospitalizations was observed in the 2020 season followed by an intensified peak in 2021 Q3 hospitalizations. In Figure 2, we provide an illustration of a delayed peak of predicted values (red line), assuming that hospital admissions that would have been predicted for 2020 Q3 and Q4 were shifted to 2021 Q2 and Q3 respectively. The outbreak peak that would have been expected in 2020 Q4 was observed in 2021 Q3 and found to be 1.5 times higher than the expected shift (red line in Figure 2). Similarly, in 2021 Q2 the number of cases (1,223) was 2.6 times higher than those predicted in a typical season (470), and five times higher if a seasonal shift is assumed (244). Cases in 2021 Q4 were lower than expected regardless of the assumption of a delayed (1.5 times lower), or a typical season (1.7 times lower). Details of the linear model of hospital admissions are in the SI.

RSV associated hospitalization rates at the state and county levels were calculated with hospital admissions with RSV diagnoses in the numerator and the population of children age four and younger as denominator. We used state population to calculate the state hospitalization rate and county population to calculate hospitalization rates in each county. State rates followed the same seasonal pattern as the number of hospitalizations (Supplementary Figure S2). A general decreasing trend over time was observed for RSV associated hospitalization rates in Q1 and an increasing trend for all other quarters (Supplementary Table S5).

We also evaluated how the RSV associated hospitalization rates calculated in this study compare to RSV associated hospitalization rates reported in the RSV-NET from the CDC. We do not expect that both approaches have the same values because the methodologies to obtain the rates are different. The RSV-NET uses data from acute-care hospitals in 58 counties in 12 states, among which Texas is not included, and the rate is calculated using laboratory-confirmed RSV-associated hospitalizations divided by the total population residing in the surveillance area. In our analysis, hospitalization rates are calculated with diagnosis (employing ICD codes) of RSV-associated hospitalizations reported from state licensed hospitals as numerator and the total population of Texas as denominator. The hospital records with RSV-associated diagnosis are spatially distributed either by state or county, thus we used the population of the state of Texas or the population in each county as denominators. The purpose of the comparison is to evaluate if the rates that are obtained with different methodologies are similar in magnitude and temporal trends. We found that the magnitude of RSV associated hospitalization rates calculated at state level in the 2018 and 2019 seasons prior to COVID-19 are comparable but not the same than those of RSV-NET. The seasonal peak in our state level rates was observed in Q4, while the peak in RSV-NET data was observed in Q1 (Supplementary Table S6). Rose et al. (2018) reported that the onset in the geographical region that contains Texas is two weeks earlier than the national seasonal onset (11). From 2018 to 2021, state and mean county-level hospitalization rates were, on average, 1.9 and 9.5 times higher than those reported in RSV-NET data (Supplementary Table S6). During the COVID-19 pandemic in 2020 and 2021, RSV-NET and our calculated hospitalization rates showed a higher discrepancy. For example, in the 2020–2021 onset of the outbreak, the mean county level rate was 43 times the level RSV-NET and the state hospitalization rate was five times the rate reported by RSV-NET. This discrepancy is expected since hospitalization rates based on ICD-codes are not specifically linked to laboratory-confirmed RSV cases leading to (under- or) overestimation compared to population-based surveillance that uses laboratory-confirmed cases.

**Figure 3:**
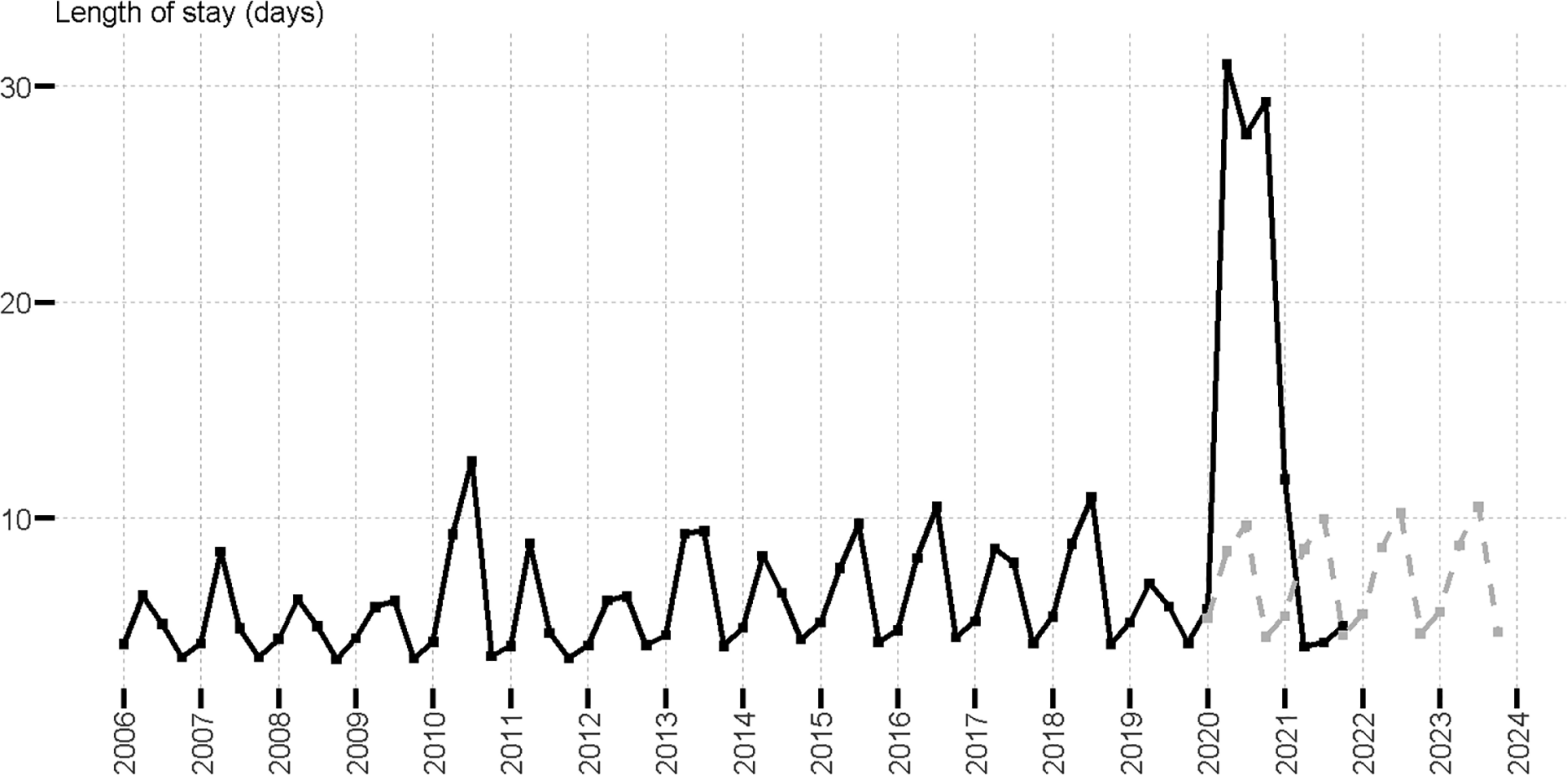
Mean length of stay of RSV hospitalizations in patients aged four years and younger; observed (black line) and predicted (gray dotted line).

We further analyzed pediatric healthcare outcomes. From 2006 to 2019, the mean length of hospital stay followed a seasonal trend with peaks of hospitalization stay duration in Q2 and Q3, and short stay duration in Q4 and Q1 (Figure 3). Figure 3 shows the observed and predicted values of the mean length of stay (obtained with linear regression models for each quarter using data from 2006 to 2019, as detailed in SI). The calculation of predicted values for 2020 to 2021 was based on the assumption that COVID-19 had no impact on length of hospital stay. Notably, we found that the mean length of hospital stay was impacted during the COVID-19 pandemic from 2020 Q2 to 2021 Q4. The mean length of stay was up to 6.5 times longer than was expected in 2020 Q4 (29.3 vs. 4.5 days). The difference between the observed mean length of hospital stay for patients with RSV in 2019 Q2 to 2019 Q4 (mean = 4.47, *n* = 7938, range 319, IQR 3) and 2020 Q2 to 2020 Q4 (mean = 29.80 *n* = 123, range 392, IQR 32.5), was statistically significant (Wilcoxon Mann-Whitney test *p* < 0.001). We also analyzed patient outcomes at the end of hospitalization and observed that COVID-19 had a relatively small impact. The overwhelming majority of patients were discharged normally (∼97%) regardless of year. However, the rate of expired status in 2020 (0.39%) was 2.28 times higher than the 2006–2019 average (0.17%) (Supplementary Table S8).

**Figure 4:**
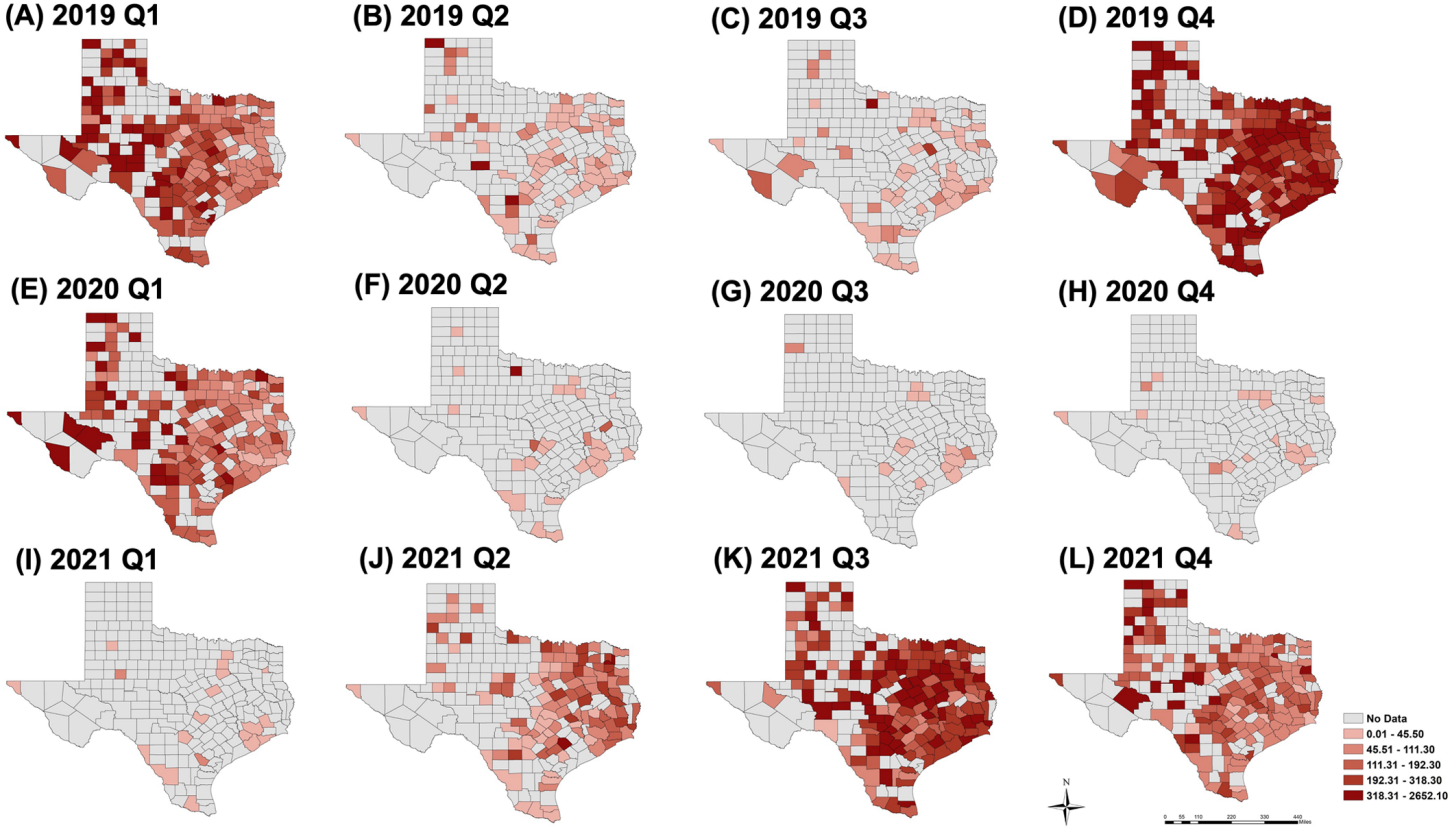
Spatial distribution of hospitalization rates per 100,000 persons at the county level for 2019 Q1 to 4 (**A–D**), 2020 Q1 to 4 (**E–H**) and 2021 Q1 to 4 (**I–L**). Year 2019 represents a “typical” season, years 2020 and 2021 show the impact of COVID-19 on the spatial distribution.

The spatial distribution of hospitalization rates at the county level across the state from 2019 to 2021 is shown in Figure 4. The year 2019 represents a typical season in which, during quarters 2 and 3, the seasonal onset appeared highest in east and south-central Texas, including the southeast border (lower Rio Grande Valley). This seasonal onset then shifted to a broader distribution across the state as cases increased in 2019 Q4 and 2020 Q1. In a subsequent analysis of 2020 Q2–2021 Q1, only a handful of counties reported RSV cases (the lowest was 14 counties in 2020 Q3). The highest hospitalization rate (490 cases per 100,000) in 2020 Q2 was observed in a rural county, while three urban counties (Bexar, Dallas, and Harris) reported cases during the pandemic (2020 Q2 to 2021 Q1). In 2021 Q2, the RSV re-emergence appears to follow a typical onset (2019 Q2) but with higher and more broadly distributed rates throughout the east of the state. In the rural area of the southwest border (without accounting for urban El Paso County) no cases were observed during and after COVID-19, but cases were reported prior to 2020 Q2.

## 4. Discussion

Tracking RSV-related diseases, especially in susceptible subgroups, is important since they represent a global economic and social burden (19, 20). Specifically in the U.S., approximately 58,000 to 80,000 children younger than 5 years old are hospitalized due to RSV annually (5). Understanding seasonality and regional factors at the state and county levels can improve the design of surveillance and prevention strategies and increase their efficacy. We analyzed trends in pediatric RSV-associated hospitalizations and evaluated changes in hospital admissions and length of stay during the COVID-19 pandemic using public use data files from the Texas DSHS.

Our results indicate that, prior to COVID-19, children 29–365 days of age were at high risk for RSV-related hospitalization. The hospitalization burden shifted to the youngest ages of hospitalized patients after COVID-19 (1–28 days of age). One possible explanation is that the expected immunity protection by maternal antibodies (21, 22) changed during 2020 and 2021. When explaining different immunity scenarios, Methi et al. (2022) hypothesized that lack of viral exposure to mothers during pregnancy can affect the vulnerability of newborn children (10). Age related shifts in hospitalization burden have been reported in three other investigations at different age group resolutions with contradictory findings (12, 13, 17). Using PCR surveillance from one hospital in New York city, Agha and Avner (2021) reported that the median age of patients changed from 17 months in the 2019 season to 6 months in 2021 (12). Another study using RSV detections in Western Australia reported that the median age of hospitalizations shifted from less than one year to a median age of 1.5 years (13). In a third study that used data from laboratory testing, hospital admissions, and syndromic surveillance in England, Bardsley et al. (2022) reported that the burden of laboratory-confirmed RSV cases shifted from children of <1 year pre-COVID-19 to 1–4 years in summer of 2021 (17). In general, results from this and other research in different settings, countries, and time periods (6, 7, 9, 23–26) have demonstrated that the highest hospitalization burden levels are commonly observed among children <1 years of age. The COVID-19 impact on shifts in hospitalization burden among children requires further study.

At present, it is well known that the seasonality and intensity of RSV hospitalizations were affected by the COVID-19 pandemic (10, 12–17). In independent studies conducted in New York city (12) and England (17), it was speculated that the seasonal delay and more severe disease observed in younger infants could be due to a lack of viral exposure during 2020. In Australia, loosening of physical distancing recommendations and border restrictions were possibly related to a higher-than-expected inter-seasonal resurgence (13). Our investigations revealed a lower number of RSV-hospitalizations during 2020, an unusual peak in the spring and summer of 2021, and an increase in hospitalizations by a factor of 1.5 in the third quarter of 2021. The impact during COVID-19 is further highlighted during the unusual RSV onset of the 2021 season that resulted in a higher than expected increase in hospitalizations by a factor of 2.6. Our data reinforce the findings of a previously published “maternal and child immunity debt” scenario in Methi et al. (2022) that assumed the COVID-19 pandemic resulted in a shift in the peak of RSV hospitalizations from the winter of 2020 to a non-typical peak in summer of 2021, and twice as many hospitalizations for children 0–12 months (10). Methi et al. (2022) studied hospital admissions of common respiratory tract infections including RSV in Norway and projected possible hospital admissions based on different immunity debt scenarios (10). They hypothesized that the social-interaction restrictions during the COVID-19 pandemic resulted in a lack of viral exposure and a consequent immunity debt for respiratory tract infections. In Texas, the first statewide COVID-19-related mandate limiting social gatherings was instituted on March 19, 2020. Large scale polices and information about the COVID-19 pandemic clearly impacted human behaviors (27). Infants who would have normally started in daycare in 2020 were perhaps delayed and did not encounter other children and gain the immunity they might have under more normal conditions. As the state reopened through a phased approach, shifts in RSV cases began to be reported in summer 2021. By the end of 2022, the CDC reported that it was still too early to predict when the former seasonal patterns might return (5). Once data are available, further assessment of trends in 2022–2023 to illuminate the complete impact of the COVID-19 pandemic on RSV is warranted. Modeling suggests that seasonal shifts and intensified RSV outbreaks may occur in coming seasons (28).

Administrative hospitalization data usually lags 1 year. For example, our analysis conducted in the year 2022 contains hospital discharge data from 2006 to 2021 but year 2022 is not available. Therefore, up-to-date hospitalization rates at state and county level cannot be obtained using hospital discharge data. On the other hand, surveillance data usually lags about one month (RSV-NET monthly rate of November 2022 was available in December 2022). A discrepancy is expected between hospitalization rates calculated with different methodologies. Specifically, we calculated hospitalization rates using hospital diagnosis (not specifically linked to laboratory test confirmation) while RSV-NET reports hospitalization rates calculated with laboratory-confirmed tests. However, if the difference between hospitalization rates obtained from population-based surveillance, such as RSV-NET, and hospitalization rates obtained from administrative data is known and consistent over time, one could estimate up-to-date hospitalization rates at the state and county levels. Our results are similar in magnitude to population-based surveillance in the U.S. (RSV-NET) and indicate that hospitalization rates in the 2021–2022 season were twice as high as expected. Based on 2018–2021 values, we can assume that state-level hospitalization rates are on average 1.9 times higher than rates derived from population-based surveillance (from Supplementary Table S6), and predict the most up to date state level rate (318 × 1.9 cases per 100,000 for 2022Q4). This analysis suggests that the winter 2022 seasonal peak could be at least twice as high as the two previous years, and the highest seen in the last 17 years (600 cases per 100,000). This value is a low estimate as surveillance of the last quarter of 2022 is still ongoing at the time of the preparation of this manuscript (population-based surveillance rates for December have not yet been reported). Additionally, quantification of the discrepancy between the two methodologies for a longer time would be needed to assess if the difference is consistent over time.

Regarding the duration of hospitalization before COVID-19, we observed a seasonal trend with longer mean hospital stays in Q2 and Q3, and shorter hospital stays in Q4 and Q1 as well as a consistent median hospital duration of three days across all four quarters. Wang et al. (2022), using RSV hospital admission data from 2001 to 2018 for children <5 years of age in seven European countries, reported a median length of stay of two to four days (8). In our study, during the COVID-19 pandemic, the overall number of hospitalizations were low, and patient stays were significantly longer. Similarly, Agha and Avner (2021) reported that the median length of stay increased by one day during the reemergence of RSV in 2021 (12).

In addition to tracking temporal variations in RSV disease rates, hospital data provides information that can be used to monitor the spatial distribution of RSV related hospitalization at more refined scales. While larger regional variation has been observed (29), geographic differences at local scales may better inform surveillance. In general, we observed that county-level hospitalization rates had a wider range, and were higher than those reported in state-level and population-based surveillance. Counties whose healthcare infrastructure is at risk of becoming overwhelmed can be detected by conducting spatial analysis at the county level. This information will be very valuable for healthcare planning.

There are some potential limitations of this type of study. First, the use of ICD codes to define an RSV case without verification by laboratory data may cause a misrepresentation of the true hospitalization burden. This misrepresentation could be attributed to differences in healthcare settings, coding, and testing practices (RSV-coded vs. RSV-confirmed), or the circulation of the infectious virus (6, 7, 26). Although hospitalization rates are difficult to compare across studies, our rates are within values reported in previously published studies that used routinely collected hospital data. Specifically, Reeves et al. (2017) reported 531 RSV cases per 100,000 in children one to four years of age in England from 2007 to 2012 (6), and Reeves et al. (2020), in a study covering seven European countries from 2001 to 2017, reported rates of 880 to 2,454 RSV cases per 100,000 in children less than 5 years of age (7). One possible interpretation is that these comparable values demonstrate that RSV hospitalization rates obtained from hospital admission datasets are consistent and useful in efforts to develop a deeper understanding of the hospital burden of RSV in children. A counter argument is that retrospective studies that use hospital diagnosis reported as ICD-codes are subject to similar bias due to misrepresentation of the true hospital burden. Further analysis of differences between hospitalization numbers using an ICD code and laboratory-confirmed RSV numbers within the same dataset would inform the magnitude of the misrepresentation. Unfortunately, the datasets used in this study do not contain information on whether an RSV-associated hospitalization diagnose was confirmed with laboratory tests. A conclusion from this limitation is that we presented a methodology that use public use datasets routinely collected by the state of Texas that can be the base for further studies. Additionally, routinely collected data on healthcare outcomes, such as length of stay, have the potential to be used to inform healthcare prevention strategies. For example, Hartmann et al. (2022) found that pneumonia, oxygen need, and preterm birth were predictors for prolonged hospital stays (25).

A second limitation of this study is that we did not examine co-infections or risk factors for RSV hospitalizations that could have impacted some of our results. It is now known that during the 2022 season of RSV, hospitals have been filled beyond capacity with cases of RSV, influenza, and COVID-19 (30). There are some interesting analyses beyond the scope of this brief research report. One could for example evaluate if changes in length of stay or other health care outcomes are associated to occurrence of co-infections. Similarly, an analysis of temporal and spatial trends of risk factors for respiratory infections, such as chronic lung disease or congenital cardiac defects could provide a deeper layer of information regarding the RSV-associated hospitalization burden.

A third limitation is that we did not analyze if the lack of viral exposure during the COVID-19 pandemic was the reason for observing an atypical peak in the 2021 RSV season and explains an immunity debt hypothesis. The analysis is out of the scope of this brief research report. However, one approach to test the immunity debt hypothesis could be to examine the use of prophylactic antibodies against RSV and associated outcomes. Another approach could be to quantify if a similar atypical RSV season is observed in other vulnerable populations, such as adults older than 65 years of age.

In summary, monitoring RSV-associated hospitalizations, especially in vulnerable subgroups like children, is important as there is a significant burden of RSV infections in pediatric hospitals. The altered seasonality and extended hospital stays reported during and after the COVID-19 pandemic highlights both the role of large-scale behavioral changes and the impact of other respiratory diseases on RSV-related hospital admissions.

## Data Availability

Publicly available datasets were analyzed in this study. This data can be found here: The data analyzed in this study was obtained from the Texas Public Use Data Files (PUDF) of the Department of State Human Services (DSHS), the following licenses/restrictions apply Data Use Agreement. Requests to access these datasets should be directed to the Texas DSHS [https://www.dshs.texas.gov/texas-health-care-information-collection/health-data-researcher-information/texas-inpatient-public-use].
