# Supplementary material for: Quantifying changes in respiratory syncytial virus—associated hospitalizations among children in Texas during COVID-19 pandemic using records from 2006 to 2021
Source: Front Pediatr. 2023 Mar 13;11:1124316. doi: 10.3389/fped.2023.1124316 (PMC10040829; doi:10.3389/fped.2023.1124316)
Supplement: Supplementary file 1 [file Datasheet1.pdf]

Supplementary Material

Table S1 Codes from ICD-9-CM and ICD-10-CM used to select respiratory syncytial virus (RSV) cases

ICD-9-CM codes for selecting RSV cases from 2006 Q1 to 20015 Q3	079.6 RSV<br>466.11 Acute bronchiolitis due RSV<br>480.1 Pneumonia due to RSV
ICD-10-CM codes for selecting RSV cases from 2015 Q4 to 20020 Q4	B97.4 RSV as the cause of diseases classified elsewhere<br>J20.5 Acute bronchitis due to RSV<br>J21.0 Acute bronchiolitis due to RSV<br>J12.1 RSV pneumonia

Table S2 Total number of RSV cases (hospital admission percentage) collected from 2006 to 2019, 2020 and 2021.

Age	2006-2019	2020	2021
1-28 days	9 982(6.97)	197 (5.08)	526 (4.42)
29-365 days	84 788 (59.24)	1 664 (42.92)	5 458 (45.83)
1-4 years	33 889 (23.68)	697 (17.98)	3 435 (28.84)
>4 years	14 475 (10.11)	1 319 (34.02)	2 490 (20.91)

Table S3 Total number of RSV cases (hospital admission percentage) collected for patients under 5 years of age from 2006 to 2019, 2020 and 2021.

Age	Q1	Q2	Q3	Q4
		Average 2006-2019		
1-28 days	395 (8.23)	26 (7.28)	12 (7.69)	278 (7.18)
29-365 days	3229 (67.27)	240 (67.23)	93 (59.62)	2492 (64.38)
1-4 years	1176 (24.50)	91 (25.49)	51 (32.69)	1101 (28.44)
		2020		
1-28 days	189 (7.76)	0 (0)	4 (16.67)	4 (9.3)
29-365 days	1589 (65.26)	42(75)	7 (29.17)	26 (60.57)
1-4 years	657 (26.98)	14 (25)	13 (54.17)	13 (30.23)
		2021		
1-28 days	20 (54.05)	682 (55.76)	3357(58.55)	1399 (57.69)
29-365 days	0 (0)	44 (3.6)	341 (5.95)	141 (5.81)
1-4 years	17 (45.95)	497 (40.64)	2036 (35.51)	885 (36.49)

Linear model of hospital admissions for each quarter

We grouped hospital admissions by calendar quarter and generated a linear regression model as follows:

$$HA_q = \beta_q + \alpha_q t \quad \text{Eq. S1}$$

where HA_q is the number of hospital admissions and t is the year (2006=1 to 2019=14), β_q is the intercept and α_q is the quarterly temporal trend.

We used the following linear model function in R: lmodel ← lm(hosp ~ t, data = DF_Q1), where hosp is the number of hospitalizations, t is the year (2006=1 to 2019=14), and DF_Q1 is the data frame containing values for quarter 1.

Results for each model are reported in Table S4. The plot of all models incorporated in an overall seasonal trend can be found in Figure S1.

Table S4 Values of the linear model coefficients for each linear model

Coefficient	value	p-value
β_1	5892.40	***
α_1	-145.51	
β_2	258.67	***
α_2	13.24	**
β_3	80.74	.
α_3	10.23	.
β_4	3620.81	***
α_4	33.67	

Significance code 0 ‘***’ 0.001 ‘**’ 0.01 ‘*’ 0.05 ‘.’ 0.1 ‘ ’ 1

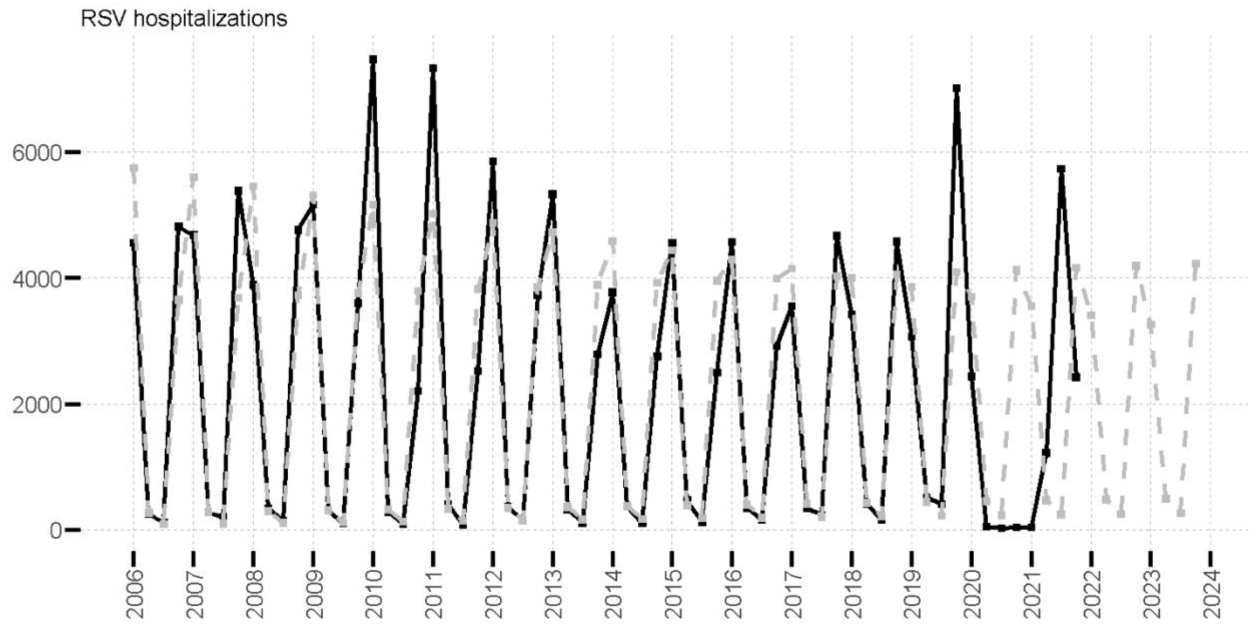

Figure S1. RSV hospitalizations, observed (black line) and predicted (gray dotted line). Values generated using Equation S1.

Linear model of hospitalization rate at state level for each quarter

We generated hospitalization rates at a state level using the population of children four years of age or younger in the state of Texas as the denominator. The hospitalization rates follow a seasonal trend similar to the hospitalization numbers. We generated a linear regression model as described in the previous section (Eq. S1). Results for each model are in Table S5 and Figure S2.

Table S5 Values of the linear model coefficients for each linear model

Coefficient	value	p-value
β_1	312.32	***
α_1	-9.13	.
β_2	14.21	***
α_2	0.50	*
β_3	4.70	*
α_3	0.42	.
β_4	198.77	***
α_4	-0.54	

Significance code 0 ‘***’ 0.001 ‘**’ 0.01 ‘*’ 0.05 ‘.’ 0.1 ‘ ’ 1

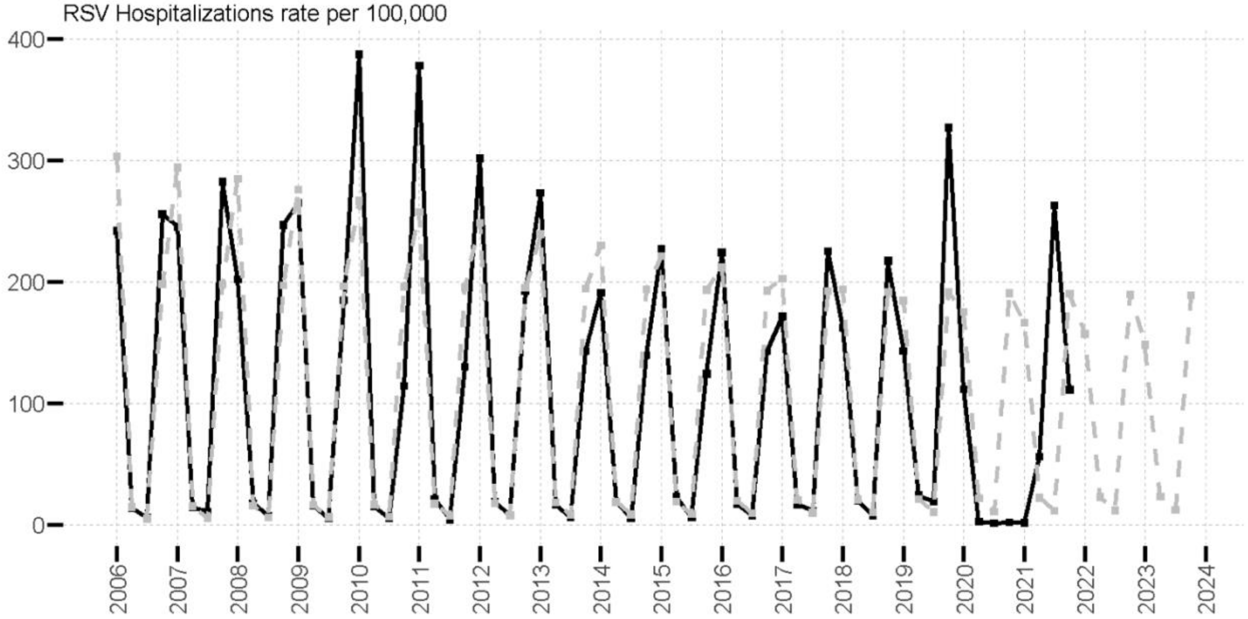

Figure S2 Hospitalization rate at the state level, observed (black line) and predicted (gray dotted line). Values were generated using Equation S1.

Table S6 Hospitalization rates (per 100,000) for patients under five years of age from RSV-NET and from our 2018 to 2021 dataset. RSV-NET does not have values for pediatric hospitalization rates prior to 2018.

Year	RSV-NET	PUDF state level	PUDF county level mean (CI)	PUDF state/ RSV-NET	PUDF county/ RSV-NET
2018		162.4	285.3(240.5-330.1)		
2018.25		19.6	51.2(36.8-65.6)		
2018.5		7.5	28.1(15.9-40.3)		
2018.75	104.1	217.3	262.2(234.2-290.2)	2.1	2.5
2019	134.4	143.1	259.1(219.8-298.4)	1.1	1.9
2019.25	9.9	24.2	66.4(46.1-86.7)	2.4	6.7
2019.5		19.1	50.4(31.0-69.8)		
2019.75	159.1	327.1	346.7(320.3-373.3)	2.1	2.2
2020	161.6	111.7	208.6(173.9-243.3)	0.7	1.3
2020.25	1.1	2.6	37.3(-1.5-76.1)	2.4	33.9
2020.5		1.1	10.9(0.8-21.0)		
2020.75	0.4	2.0	17.3(4.8-29.8)	5.0	43.3
2021	1.9	1.7	10.8(3.6-18.0)	0.9	5.7
2021.25	30.8	56.1	113.2(96.3-130.1)	1.8	3.7
2021.5	144.3	263.0	310.9(280.0-341.8)	1.8	2.2
2021.75		111.2	198.5(167.6-229.4)	0.7	1.3
2022	31.2				
2022.25	35.2				
2022.5	95.7				
2022.75	318.0				

Linear model of length of stay for each quarter

We generated statistical descriptions for the variable length of stay (LOS). For years 2006 to 2019 the median LOS was three days, which is consistent across quarters and years. However, LOS exceeded the median at six days in 2020 Q2 and 3.5 days in 2020 Q3. LOS decreased to two days in 2021 Q1. The LOS mean overall, however, followed a seasonal trend. Therefore, we generated a linear regression model as described in the previous section (Eq. S1). Results are in Table S7 and Figure S3.

Table S7 Values of the linear model coefficients for each linear model

**Coefficient**	**value**	**p-value**
β_1	3.8908	***
α_1	0.0981	***
β_2	7.0885	***
α_2	0.0917	
β_3	5.431	**
α_3	0.283	
β_4	3.3463	***
α_4	0.0763	***

Significance code 0 ‘***’ 0.001 ‘**’ 0.01 ‘*’ 0.05 ‘.’ 0.1 ‘ ’ 1

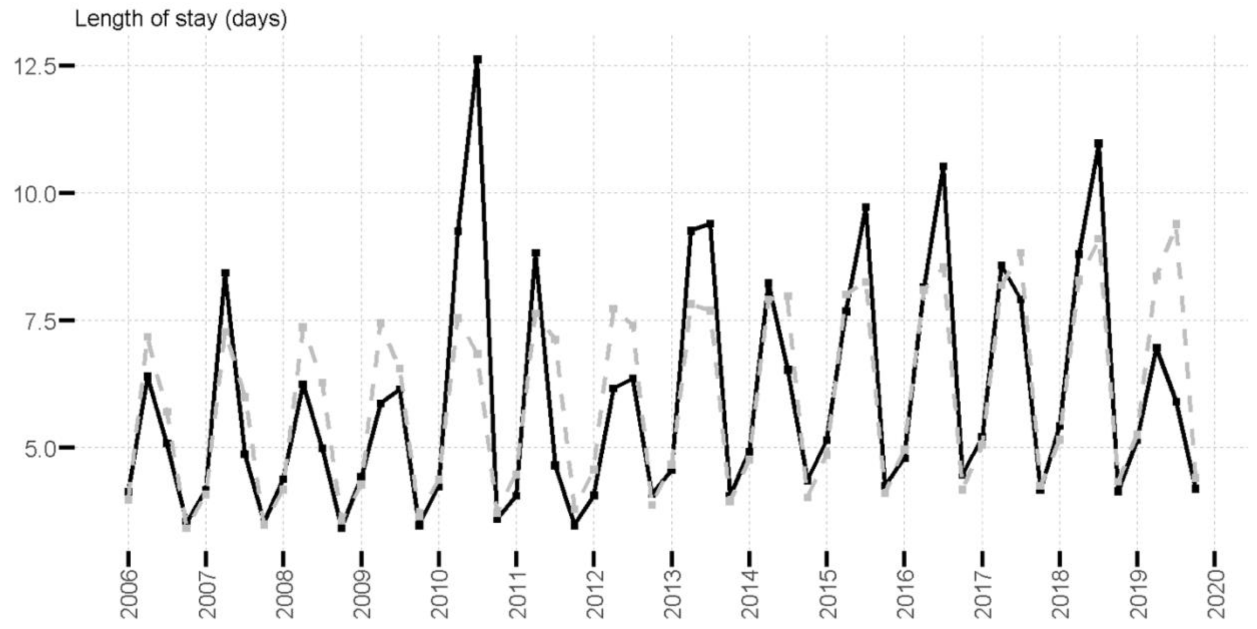

Figure S3. Length of stay (LOS), observed (black line) and predicted (gray dotted line). Values generated using Equation S1.

Table S8- Percentage of RSV cases (RSV discharge type/RSV hospitalizations) for patients five years of age or under grouped by status at the end of service (outcome).

Year	Normal discharge	Expired	Left against medical advice	Still a patient	Hospice	Other
2006	96.99	0.12	0.08	0.02		2.78
2007	97.29	0.13	0.08	0.08	0.01	2.42
2008	97.12	0.19	0.08	0.18	0.07	2.37
2009	97.14	0.14	0.11	0.09	0.12	2.40
2010	97.43	0.19	0.07	0.01	0.02	2.29
2011	97.16	0.14	0.06	0.01	0.05	2.59
2012	96.97	0.16	0.07	0.04	0.04	2.73
2013	97.26	0.22	0.02	0.19	0.02	2.28
2014	96.84	0.21	0.11	0.10	0.01	2.71
2015	97.32	0.25	0.05	0.05	0.04	2.29
2016	96.96	0.14	0.06	0.10	0.04	2.70
2017	96.86	0.24	0.10	0.02	0.01	2.76
2018	96.72	0.14	0.08	0.02	0.02	3.01
2019	96.98	0.14	0.11		0.07	2.70
2020	96.01	0.39	0.20			3.40
2021	97.15	0.13	0.12	0.03	0.04	2.53
**Average**	97.00	0.19	0.09	0.07	0.04	2.62
